# Occupational Safety and Related Impacts on Health and the Environment

**DOI:** 10.3390/ijerph13100988

**Published:** 2016-10-05

**Authors:** Andrew Watterson

**Affiliations:** Occupational and Environmental Health Research Group, Faculty of Health Sciences and Sport, University of Stirling, Stirling FK9 4LA, UK; aew1@stir.ac.uk; Tel.: +44-1786-466-283

**Keywords:** occupational safety and health, environmental health, public health and safety

## Abstract

The inter-relationship between safety, health and the ‘environment’ is a complex and at times a relatively neglected topic. In this issue, ‘safety’ is often viewed by contributors as ‘health and safety’ and includes occupationally-related ill health as well as injury or harm to employees and the wider public. ‘Environment’ is also interpreted in the widest sense covering both physical and work environments with upstream work hazards presenting risks to downstream communities. The focus is very much on exploring and where possible addressing the challenges, some old and some facing workers in a range of public and private settings and also at times their nearby communities. The 19 papers in the issue cover public and private sectors, global and very local populations, macro-theoretical perspectives, large epidemiological and some single factory or hospital site small case studies. A number of the papers are just beginning to explore and draw out for the first time the risks from hazards in their part of the world. The methodologies adopted also range from lab-based studies through ergonomic assessments and interventions to therapeutic approaches.

All the papers served to shed light on situations that exist in workplaces internationally where work environments confront tensions between production and workloads, funding, staffing and worker and community health and safety. These conflicts between safety and ‘profit’ in the private sector or service delivery in the public sector remain perennial [1].

A macro-theoretical context for the issue is offered by Beck who locates his paper within debates about risk, globalism and neoliberalism based on his substantial analysis of 105 major industrial incidents between 1971 and 2000 [2]. As he points out, “reviewing qualitative evidence on the impact of structural adjustment reforms in industrialising countries, the export of waste and hazardous waste recycling to these countries and new patterns of domestic industrialization suggests that workers in industrialising countries continue to face far greater levels of hazard exposure than those of developed countries”. This analysis is partly confirmed by papers from Africa and Asia (but important health and safety challenges are still clearly present in Europe and Australia as other papers in the issue demonstrate). The study by Hinson et al. reveals how exposures to ‘old’ and well-known hazards continue to present risks and cause diseases in new populations of textile workers in countries such as Benin where occupational health research in many respects is nascent [3].

In the private sector the continued risk run by petrol station employees to well established hazards such as the additive methyl tert-butyl ether, no longer used in petrol in many other countries, is examined by Hu et al. in the Chinese context [4]. The authors carried out a health risk assessment of 97 petrol attendants using USA Environmental Protection Agency methodologies. In Portugal, Viegas et al. not only explore fungal disease in slaughterhouses, linked to biological organisms suspended in the air—a bioaerosol—exposure as well as more direct contact with animal matter, but also offer a protocol to ensure occupational exposures to fungal contamination are better characterised in the future [5]. The physical and ergonomic problems faced by poultry abattoir workers are reviewed in South Africa by Marmse et al. who provide a quick reference guide to hazards and related risk for use by industry, workers and health professionals with a view to improving safety management of occupational health in the sector [6]. The physical workload of male workers in the construction industry in Norway is investigated by Lunde and his colleagues who describe the cardiovascular burden of 255 such workers [7]. Construction workers in Chinese high altitude tunnels face a different set of risks and Guo and colleagues looked at the oxygen supply standards which might apply when workers are engaged in heavy physical work in tunnels [8]. Importantly the authors moot a standard for oxygen supply to this workforce.

Sometimes similar and sometimes more subtle and unexpected challenges to health and safety exist within the ‘public sector’ workplaces that feature in this issue. Ergonomic, work organisation and management and related psychosocial problems may dominate in some sectors and some jobs. In Germany, the noise exposures of a neglected group of nursery school teachers were measured by Gebauer and colleagues [9]. They identified the dropping of toy bricks into metal storage cases as a major source of high noise levels and offered noise reduction solutions through putting polyurethane foam inserts into the cases. In Thailand, researchers wanted to apply an intervention to ergonomic problems that had been described in Thai hospital orderlies [10]. Chanchai and colleagues used a participatory ergonomics approach as well as a randomised control trial and the application of standard questionnaires with 100 orderlies. Positive results followed the work environment interventions especially in reducing physical work environment risk factors for musculoskeletal disorders and promoting positive factors. Rather different ergonomic factors were assessed in a study of Australian police recruits and their training by Orr and colleagues [11]. The relationship between leg power and reported injuries and illnesses in a cohort of 1021 recruits was analysed showing recruits with weaker leg power had a significantly greater risk of injury and disease during basic recruit training.

More subtle effects of work organisation and worker health were teased out in a study from Lithuania looking at nurses’ well-being linked to arts activity [12]. Karpavičiūtė and Macijauskienė took a cohort of 115 staff over 10 weeks, developed an arts intervention and used standard health questionnaires to evaluate impacts. Their findings suggest arts activity promoted nursing staff well-being at work. Whilst another study by Zhao et al. in China looked at the direct effects of violence at work on hospital staff in 19 hospitals in 6 cities using a theoretically informed approach based on social support theory [13]. The study highlighted the need for more support for vulnerable staff at both organisational and societal levels. Still in hospital settings and again in China, the monitoring needs of nuclear medicine staff in a specialized hospital were the focus of a small study of 18 staff by Wang et al. [14]. The study drew on internal agency criteria and identified the need for monitoring of staff.

Particular and relatively unusual and high risk safety and health hazards exist for workers in some sectors: for example, in the fishing, maritime and related industries. Naval personnel face a range of risks. Kan and colleagues looked at how knowledge of jelly fish sting hazards affected 120 Chinese sailors by using a questionnaire [15]. Whilst stings were relatively common, the knowledge base of the sailors was not extensive and the study found better education and training were needed to address this problem. Training and information were also key issues in a survey of hypothermia in the Chinese navy among 111 navy members conducted by Li and others [16]. The results suggested the recognition and treatment of accidental hypothermia at that time were inadequate in the Chinese Navy and extensive education programmes were needed on the subject.

Epidemiological and environmental studies can either confirm existing problems or lead to the recognition of new health and safety problems. An epidemiological study across 1055 patients of eye injuries in China by Cai and Zhang using the World Health Organization International Classification of Diseases and other classifications found over 40% were occupationally-related [17]. The study therefore provides a baseline for future work on the subject and most importantly for the development of prevention strategies. An environmental study by Chen and others explored the particulate exposure of staff in a police station near a major road in Taiwan at different times of day, with different traffic volumes and vehicle types and with different weather conditions [18]. Few studies had been done on this occupational group and the researchers found high polycyclic aromatic hydrocarbons concentrations at certain times which led them to conclude ‘special attention needs to be given to protect police officers from exposure to high particulate matter concentration’. Tong et al. examined the possible genetic effects of dimethyl formamide on a Chinese population. This is important as China produces and consumes the largest quantities of DMF in the world [19]. The researchers found genetic variations may be associated with DMF-induced abnormal liver function in the Chinese Han population. Finally, an Italian study by Carducci used quantitative microbial risk assessment, developed in the past for drinking water and food safety studies, to examine occupational risks associated with human adenovirus infection [20]. The researchers considered the approach a novel one in such settings and, although cautious, considered it merited further investigation. 
